# A Computerized Order Entry System Was Adopted with High User Satisfaction at an Orthopedic Teaching Hospital

**DOI:** 10.1007/s11420-013-9377-1

**Published:** 2014-01-09

**Authors:** Mary Murray-Weir, Steven Magid, Laura Robbins, Patricia Quinlan, Pamela Sanchez-Villagomez, Steven H. Shaha

**Affiliations:** 1 Hospital for Special Surgery, 535 East 70th Street, New York, NY 10021 USA; 2 Danbury Hospital, 24 Hospital Avenue, Danbury, CT 06810 USA; 3 Allscripts, Three Ravinia Drive, Atlanta, GA 30346 USA; 4 Rehabilitation Department, Hospital for Special Surgery, 535 East 70th Street, New York, NY 10021 USA

**Keywords:** satisfaction survey, computerized prescriber order entry, potential errors, implementation, multidisciplinary

## Abstract

**Background:**

Computerized provider order entry (CPOE) has been considered essential for the reduction of medical errors and increased patient safety. Assessment of staff perception regarding a CPOE system is important for satisfaction and adoption. Incorporation of user feedback can greatly improve the functionality of a system and promote user satisfaction.

**Questions/Purposes:**

This study aims to develop an informatics staff satisfaction survey instrument and to understand what components of computerized prescriber order entry (CPOE) contribute to staff satisfaction and its variability over time.

**Methods:**

The 22-question survey was developed by a multidisciplinary group and focused on patient data including demographics, orders, medications, laboratory, and radiology data. The questions were designed to understand if clinicians (1) could easily access the information needed to properly take care of patients, (2) could act upon the information once acquired, (3) could obtain the information clearly, and (4) were alerted to potential errors. The survey was distributed just prior to “go-live,” 6 and 12 months after go-live. Responses were given on a five-point Likert scale.

**Results:**

The survey results post-implementation showed user satisfaction with CPOE. Satisfaction regarding the ease of obtaining orders, medication, and lab data had a significant improvement at 6 and 12 months post-implementation, *p* < 0.001. Satisfaction that the computerized order entry system provided information needed to take care of their patients improved, *p* < 0.01. At 1 year post-implementation, user satisfaction declined from 6 months earlier but still demonstrated an overall increase in satisfaction from pre-implementation.

**Conclusion:**

Compared prior to go-live, clinicians are satisfied or very satisfied across multiple spheres and multiple disciplines. At all time points, clinicians were able to obtain information required to take care of their patients. However, post-go-live, it was easier to obtain and act upon as well as more clear and understandable.

**Electronic supplementary material:**

The online version of this article (doi:10.1007/s11420-013-9377-1) contains supplementary material, which is available to authorized users.

## Introduction

The implementation of information technology in health care has been regarded as essential to the reduction of medical errors and increased patient safety. The 1998 report of the Institute of Medicine (IOM), *To err is human: building a safer health system*, estimated that 44,000 to 98,000 people die annually in the USA from medical errors [1]. The IOM’s subsequent report, *Crossing the quality chasm*, promotes the use of computerized information systems to improve patient safety regarding medical errors [2].

Computerized provider order entry (CPOE) systems allow health-care providers to directly enter orders for patient care into an automated system. Benefits include the elimination of transcription errors, rapid data retrieval, improved communication and response time, and clinical decision support. These benefits add up to an improved quality of patient care and decreased health-care costs.

Despite the benefits of using a CPOE system, there are a number of barriers to implementation. Cost to develop and install a system can be a major obstacle. Estimated costs to implement a system at a 500-bed hospital without network upgrades is $8 million, with ongoing maintenance costs of more than $1 million a year [1]. Another widely recognized barrier is user acceptance, with studies across the USA demonstrating that physicians are particularly reluctant to use CPOE [3].

User satisfaction with CPOE is a predictor of compliance with CPOE use [3]. An important consideration is to be familiar with the users of the system. Researchers in a study by Murff and Kannry found that user satisfaction correlated with a well-designed user interface, suggesting the importance of designing the system with a user in mind [1]. Incorporation of user feedback can greatly improve the functionality of a system and promote user satisfaction. Previous researchers who examined clinicians’ perceptions of CPOE implementation found that there is a clear difference between clinicians’ satisfaction with custom made versus commercially available systems [3]. The “commercial off-the-shelf” systems are not developed with any individual location in mind, potentially negatively impacting the usability for the system and acceptance by users [4]. In order to assess user satisfaction, an informatics survey instrument was developed to assess important aspects critical to patient care:

1. Could the clinician easily access information needed for proper patient care,
2. Could the clinician act upon patient information once acquired,
3. Was the information clear and understandable, and
4. Was the clinician adequately informed of potential errors or safety problems.

Prior to implementation of this system, a CPOE system did not exist at Hospital for Special Surgery (HSS). Staff primarily relied on paper communication for orders, medical administration records (MARs), and patient demographics (i.e., vital signs, allergies, and height and weight), and paper and limited electronic communication was available for laboratory and radiology results (see Table 1). Computerized access to information also varied from provider to provider.

**Table 1:** Access to patient data pre- and post-CPOE implementation

Pre-system implementation	Post-system implementation
No CPOE	100% CPOE
Paper communications for	Went live electronically with
- Orders	- CPOE
- MAR	- eMAR
- Vital signs	- Flow sheets: vital signs, allergies, height, and weight
- Allergies	
- Height and weight	- Lab and radiology results
Electronic and paper communication for	
- Lab and radiology results	
Providers had limited/varied computerized access to patient demographics and laboratory and radiology results	All providers can access patient demographics and laboratory and radiology results electronically

Observation of the adaptation of CPOE for the care of postoperative total joint patients at another institution identified multidisciplinary authoring as a challenge [5], and the efforts of the different populations of clinicians need to be considered [4]. Customization of the HSS system was an interdisciplinary process. Involvement of key members of the health-care team was important in the development of the system to ensure inclusion of all aspects of care. To build staff support and gain input, an interdisciplinary development team was formed, and meetings were conducted to discuss customization options. Ongoing support and training were provided by Eclipsys throughout the process. This study was performed to assess user satisfaction with CliniCIS, a CPOE system initiated last July 2007 at HSS. The study also assessed staff perception regarding the impact of the new system on patient safety and potential errors.

## Methods

A survey was conducted pre-implementation and at 6 and 12 months post-implementation to evaluate user perceptions of health information management.

A custom survey was created for assessing satisfaction, primarily because none was available that was capable of reflecting HSS’s multidisciplinary approach to care as well as differences in satisfaction between provider groups. In addition, there was a challenge to find methods to administer the survey that would maximize participation from the staff due to variations in computer access and computer skills of the staff.

The “patient information systems survey” was developed by a multidisciplinary workgroup consisting of a physician, nurse, physical therapist, and medical educator with a background in survey techniques. The survey consisted of 22 questions using a five-point Likert scale and an open-ended comment section. Questions focused on patient data common to all staff (demographics, orders, medications, and laboratory and radiology results) and were designed to ascertain staff perception of the available data.

Demographically, nursing, rehabilitation, and orthopedics represented a large majority of the respondents to the survey at each point in time (Table 2). Approximately 70% of those returning the survey had worked in another hospital prior to working at HSS, with roughly half of those institutions having a computerized physician order entry system for accessing patient information.

**Table 2:** Survey respondents

Demographics of respondents	Pre-implementation	6 months post-implementation	1 year post-implementation
Years worked at HSS			
<1 year	38	30	32
1–5 years	90	99	86
5–10 years	39	53	37
10–15 years	25	25	15
15–20 years	18	17	14
20+ years	32	29	29
**Total**	**242**	**253**	**213**
Specialty			
Orthopedics	50	47	45
Rheumatology	15	12	14
Nursing	75	98	76
Rehab	66	45	43
Pharmacy	7	7	2
Other	19	32	27
**Total**	**232**	**241**	**207**
Position			
Attending	19	27	33
Fellow	4	8	11
Resident	2	3	3
PA	23	10	1
Nurse	90	92	67
Rehab	67	49	45
Pharmacist	5	4	1
Nutrition	3	6	4
Lab	6	10	6
Case manager	0	4	4
Radiology	1	2	2
Worked in other hospitals			
Yes	177	174	147
No	61	71	66
If yes, was there a computerized physician order entry for accessing patient’s information?			
Yes	88	90	81
No	84	78	65
Not sure	6	6	4
Did system in your previous hospital meet your needs?			
Yes	146	193	175
No	15	8	14
Not sure	85	18	5
Have the knowledge necessary to use the current system at HSS?			
Yes	146	193	175
No	15	9	14
Not sure	33	9	18

### Survey Administration and Participation

The patient information systems survey was administered three times: pre-go-live of the system, 6 months post-go-live, and 12 months post-go-live. All CliniCIS users received a survey during each 2.5-week survey period; at each of the three time points, approximately 1,200 system users were surveyed. The survey was made available via the hospital’s existing in-house electronic e-Learning system, online electronic SurveyMonkey®, and paper-based survey instruments. Respondents addressed satisfaction with the “current system” by rating their agreement with the survey question (1 = strongly disagree, 5 = strongly agree). An overall mean score was calculated for each question on the survey.

Statistical analyses conducted to discern significant differences between measurement occasions (i.e., pre vs. 6 months vs. 1 year) were ANOVAs, selected due to greater robustness versus *t* tests for inequality in standard deviations between occasions. All significance levels were set at a minimum of *p* < 0.05. Statistical analyses were conducted using SPSS version 17.0 (SAS for confirmatory purposes).

## Results

The mean score for the individual survey questions and the significant difference between pre-implementation of the computerized order entry system and at 6 months and 1 year post-implementation was calculated (Table 3).

**Table 3:** Survey questions

Question (strongly agree to strongly disagree)	Mean			Mean	
	Pre-implementation	6 months post-implementation	Significance (*p*)	1 year post-implementation	Significance (*p*)
Current system provides easy way to obtain					
Demographic data	3.55	3.96	<0.001	3.79	<0.01
Orders	3.23	4.20	<0.001	4.09	<0.001
Medications	3.07	4.07	<0.001	3.90	<0.001
Lab data	3.27	4.28	<0.001	4.14	<0.001
Radiology	3.12	4.05	<0.001	3.88	<0.01
Satisfied with accessibility of computer hardware during my workday at HSS	3.36	3.84	<0.01	3.71	<0.01
Current system facilitates work flow	3.14	3.95	<0.01	3.74	<0.01
When I get patient info I need from current system, I can easily act upon (respond to) it					
Demographic data	3.64	3.82	ns	3.75	ns
Orders	3.34	4.04	<0.01	3.96	<0.01
Medications	3.26	3.98	<0.001	3.82	<0.01
Lab data	3.36	4.15	<0.001	3.99	<0.001
Radiology	3.25	3.92	<0.01	3.76	<0.01
Current system presents patient information in a clean and understandable manner					
Demographic data	3.67	3.88	<0.05	3.75	ns
Orders	3.21	3.96	<0.01	3.81	<0.01
Medications	3.04	3.91	<0.01	3.73	<0.01
Lab data	3.26	4.15	<0.001	4.00	<0.01
Radiology	3.15	3.95	<0.001	3.75	<0.01
Current system provides me with information I need to take care of my patient	3.45	3.94	<0.01	3.84	<0.01
Current system supports efficient transfer of info from one health-care provider to another	3.06	3.70	<0.01	3.56	<0.01
Current system adequately informs me of potential errors with respect to					
Orders	2.75	3.49	<0.01	3.32	<0.01
Medications	2.73	3.61	<0.01	3.43	<0.01
Drug interactions	2.83	3.67	<0.01	3.41	<0.01
Legibility	2.58	3.95	<0.01	3.99	<0.01
Patient identification	3.19	4.02	<0.01	3.87	<0.01
I would recommend our current system to other hospitals/health-care providers	2.87	3.82	<0.01	3.63	<0.01

*ns* not significant

The survey results 6 months post-implementation showed user satisfaction with the computerized order entry system. Satisfaction regarding the facilitation of work flow of the current system improved (3.95 vs. 3.14, *p* < 0.01). Satisfaction that the computerized order entry system provided information needed to take care of their patients (3.94 vs. 3.45, *p* < 0.01) was also improved. Respondents also indicated that were more likely to recommend the current system to other hospitals and health-care providers (3.82 vs. 2.87, *p* < 0.01). When assessed at 1 year post-implementation of the computerized order entry system, user satisfaction declined from the survey administered 6 months earlier but still demonstrated an overall increase in satisfaction from pre-implementation of the system.

One of the purposes for administering the survey is to assess staff perception regarding the quality of information provided through the CPOE system. Two areas of the system that were closely addressed were prescriber orders and medications.

Prior to implementation of the CPOE system, 51.4% of survey respondents agreed or strongly agreed that the current system for communication provided an easy way to obtain orders. At 6 and 12 months post-go-live of CliniCIS, 87.1% and 84%, respectively, agreed or strongly agreed that the new system provided an easy way to obtain orders.

**Fig. 1:**
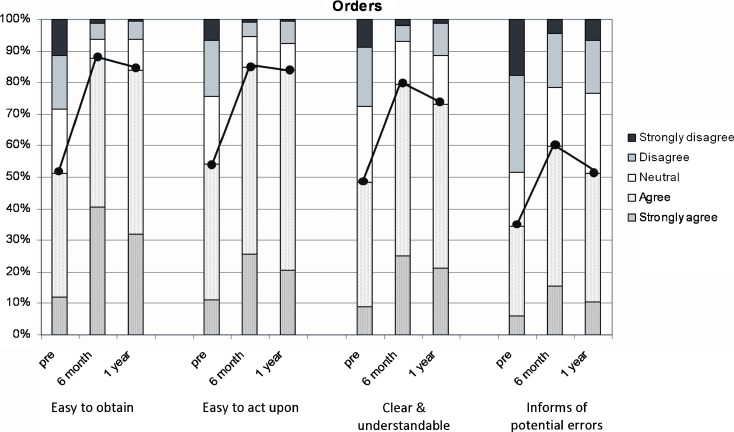
Orders.

When queried about acting on/responding to orders, 54.1% of survey respondents agreed or strongly agreed that they could easily act upon the information they obtained from the pre-go-live system. At 6 and 12 months post-go-live, 87.2% and 83.4%, respectively, agreed or strongly agreed that they could easily act upon the orders from CliniCIS (Fig. 1).

Of survey respondents, 48.3% of respondents agreed or strongly agreed that the pre-go-live system presented orders in a clear and understandable manner. Post-go-live of CliniCIS, 87.1% and 84% at 6 and 12 months, respectively, agreed or strongly agreed that the new system presented information in a clear and understandable manner. Only 34.5% of survey respondents agreed or strongly agreed that the pre-go-live system adequately informed them of potential order errors. At 6 and 12 months post-go-live of CliniCIS, 59.3% and 50.9%, respectively, agreed or strongly agreed that they were adequately informed of order errors by the new system.

Regarding the ease of obtaining medication information, only 42.8% of survey respondents agreed or strongly agreed that it was easy utilizing the pre-go-live system. Following CliniCIS implementation, 70.9% and 75.3% at 6 and 12 months post-go-live, respectively, agreed that it was easy to obtain medication information using the new system.

Upon obtaining medication information, 51.0% of survey respondents agreed or strongly agreed that they could easily act upon/respond to the information received utilizing the pre-go-live system. At 6 and 12 months post-implementation, 81.4% and 74.7%, respectively, agreed or strongly agreed that they could easily act upon/respond to medication information using the new system.

Only 41.3% of survey respondents agreed or strongly agreed that the pre-go-live system presented medication information in a clear and understandable manner. At 6 and 12 months post-go-live of CliniCIS, 76.3% and 68.7%, respectively, agreed or strongly agreed that the medication data from CliniCIS was clear and understandable.

**Fig. 2:**
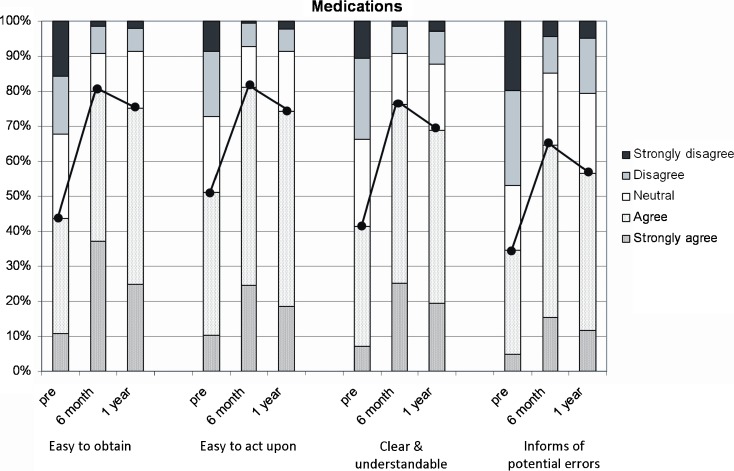
Medications.

Only 31.6% of survey respondents agreed or strongly agreed that the pre-go-live system adequately informed them of potential medication errors. At 6 and 12 months post-go-live of CliniCIS, 64.2% and 56.9%, respectively, agreed or strongly agreed that they were adequately informed of medication errors by the new system (Fig. 2).

### Open-ended Comments

Respondents provided feedback, ideas, and suggestions by an open-ended comment section that was presented at the end of the survey. Three questions were asked:

- What do you like most about the current system?
- What would you change about the current system?
- Is there anything else you would like to tell us that would help us improve the current system?

Comments were obtained for each time point for the survey administration (pre-go-live of the system, 6 months post-go-live, and 12 months post-go-live), utilizing qualitative methodology. Comment responses were categorized into common themes.

#### Pre-Go-Live

The following are the data when respondents were queried about what they like most about the current system pre-implementation of CliniCIS:

- Of the comments, 36.94% were related to the features of the current system.
  - Of these comments, 58.54% referred to aspects of the system’s functionality.
  - Approximately 31.71% mentioned accessibility to clinical information.
  - Additional comments related to technology (“latest technology,” “It’s a Windows-based point and click environment”) and validity of documentation (“written documentation backed up with personal signature…elimination of false identification”)
- Of those answering the question, 26.13% found the current system to be easy to use/user-friendly, with a quarter of these respondents stating that they felt comfortable and familiar with the system.
- Remaining comments indicated that there are just as many respondents did not like as well and were neutral about the current system (15.32% each).

The following are the data when respondents were asked about what they would change about the current system:

- Of the comments, 60.14% were related to the features of the current system.
  - Of these comments, 41.57% addressed specific functionality of the system.
  - There were 13.48% of respondents who mentioned accessibility of clinical information as well as the ability to access the system from multiple locations.
- Of them, 14.86% and 13.51%, respectively, were neutral or had nothing they would change.
- Approximately 6.08% of respondents would change “everything” about the current system.

Additional comments to help improve the current system included suggestions regarding the functionality of the current system. Responses also mentioned training on the current system as well as interfacing with other existing hospital systems.

#### Six Months Post-Implementation

The following are the data when respondents were queried about what they like most about the current system 6 months post-implementation of CliniCIS:

- Of the comments, 75.00% were related to the features of the current system. This represents an increase in satisfaction in the features of the new CPOE system over the previous system.
  - Of these comments, 27.03% referred to aspects of the system’s functionality.
  - Approximately 41.44% mentioned accessibility to clinical information.
- Of those answering the question, 12.14% found the current system to be easy to use/user-friendly.
- Of those answering the questions, 3.38% liked everything.
- Remaining comments indicated that respondents did not like and were mixed or neutral about the current system (4.73%, 3.38%, and 2.03%, respectively)

When respondents were asked about what they would change about the current system 6 months post implementation of CliniCIS, the following were obtained:

- Of the comments, 72.54% were related to the features of the current system.
  - Of these comments, 64.08% addressed specific functionality of the system.
  - Of them, 8.74% mentioned accessibility of clinical information as well as the ability to access the system from multiple locations.
  - Approximately 2.91% mentioned integration of the system with other existing systems.
  - Additional comments related to….
- Of respondents, 4.93% would change the ease of use.
- Of them, 5.63% and 13.38%, respectively, were neutral or had nothing they would change.
- Approximately 0.70% of respondents would change everything about the current system.

Suggestions for improvements of the current system were similar to the ones received at the pre-go-live implementation of the survey; functionality, training, and integration with the other existing systems remained to be the most frequently mentioned suggestions.

#### One Year Post-Implementation

When respondents were queried about what they like most about the current system 1 year post-implementation of CliniCIS, the data were as follows:

- Of the comments, 63.87% were related to the features of the current system. Although satisfaction with the new system remains high, there is a slight decrease from the users’ perceptions 6 months earlier.
  - Of these comments, 34.21% referred to aspects of the system’s functionality.
  - Of them, 44.74% mentioned accessibility to clinical information.
- More respondents (14.29%) found the current system to be easy to use/user-friendly.
- Approximately 6.73% of those answering the question commented on the efficiency/effectiveness of the system.
- Fewer respondents (1.68%) liked everything.
- Remaining comments indicated that more respondents did not like and were mixed or neutral about the current system (4.20%, 5.04%, and 4.20%, respectively) since the previous survey.

When respondents were asked about what they would change about the current system 1 year post-implementation of CliniCIS, the following are the data obtained:

- Of the comments, 72.88% were related to the features of the current system.
  - Of these comments, 38.37% continued to address specific functionality of the system.
  - Approximately 11.63% mentioned accessibility of clinical information as well as the ability to access the system from multiple locations.
  - An increasing number of respondents (13.95%) mentioned integration of the system with the other existing systems.
- Fewer respondents (3.39%) would change the ease of use.
- More respondents were neutral or had nothing they would change (5.93% and 13.56%, respectively)
- Of respondents, 0.85% would change everything about the current system.

Improvements in the functionality of the current system remained to be the most frequently mentioned suggestion by survey respondents, followed by training with the use of the system. More comments were noted regarding accessibility of the system as an improvement. Unlike the previous two administrations of the survey, integration with existing hospital systems was mentioned less frequently as a suggested improvement.

## Discussion

Implementation of the CPOE system at HSS, contributed to the overall satisfaction of clinicians at the Hospital. When surveyed at three time points users were mostly satisfied or very satisfied with the new system.

At all time points, users reported ability to obtain the information required to take care of their patients. When surveyed post-go-live, respondents expressed that the information was easier to obtain and act upon. The information was also more clear and understandable. Overall satisfaction with efficiency and work flow improved with the CPOE system.

At pre-go-live of CPOE, there was a low level of agreement by clinicians that the previous system adequately informed users of potential errors. Legibility of information and orders was also a source of dissatisfaction. The benefits of CPOE – improved communication and information about potential medical errors and transcription errors – were reported.

The initial training experience and use of the system allowed users to develop familiarity and comfort with the system. When queried 6 months post-implementation of the system, survey responses initially indicated a high level of agreement that they had the knowledge necessary to use CPOE. At this time point, overall user satisfaction was highest.

The survey administered at 1 year post-implementation showed that respondents indicated a lower level of agreement about having the knowledge necessary to use the system, as well as a subsequent decline in user satisfaction. Previous studies have also shown that user satisfaction with CPOE systems decrease with the higher levels of training, independent of prior use of CPOE or specialty [2]. We believe that this fall-off in satisfaction is related to an increasing comfort level with the system, and an increasing desire to add functionality.

Comments provided by respondents at both 6 month and 1 year post-implementation expressed consistent suggestions to improve features of the system, including functionality, accessibility and integration with other systems. As system familiarity sets in with the staff, their needs to achieve user satisfaction have evolved since the initial implementation.

Overall, the implementation of CliniCIS has been successful at HSS. Feedback from clinicians has shown the new system has been well received, with these assessments showing overall improved satisfaction by users as compared to the pre-implementation, primarily paper-based system. This success has spurred our clinicians’ interest with computerization, driving them to request a complete EMR including features such as electronic documentation, prescription writing and medical reconciliation. These findings present an important contrast and data-driven rebuttal to recently published studies that made blanket conclusions regarding the inefficacy of electronic health records, primarily because CliniCIS represents such a robust and capability-rich application whose clinical decision support does not rely on simple alerts alone.

## Electronic Supplementary Material

Below is the link to the electronic supplementary material.

ESM 1 (PDF 1224 kb)

ESM 2 (PDF 1224 kb)

ESM 3 (PDF 1209 kb)

ESM 4 (PDF 1224 kb)

ESM 5 (PDF 1224 kb)

ESM 6 (PDF 1224 kb)
